# Addressing Domestic Violence in Antenatal Care Environments in Nepal (ADVANCE) – study protocol for a randomized controlled trial evaluating a video intervention on domestic violence among pregnant women

**DOI:** 10.1186/s12889-023-16685-6

**Published:** 2023-09-15

**Authors:** P. Chalise, P. Manandhar, J. J. Infanti, J. Campbell, L. Henriksen, S. K. Joshi, R. Koju, K. D. Pun, P. Rishal, M. R. Simpson, E. Skovlund, K. Swahnberg, B. Schei, M. Lukasse

**Affiliations:** 1 Department of Public Health and Nursing, Faculty of Medicine and Health Sciences, Norwegian University of Science and Technology, Trondheim, Norway; 2 Department of Nursing and Midwifery, Kathmandu University School of Medical Sciences, Dhulikhel, Nepal; 3 Department of Community Medicine, Kathmandu Medical College, Kathmandu, Nepal; 4 Department of Community-Public Health, Johns Hopkins University School of Nursing, Baltimore, USA; 5 Department of Nursing and Health Promotion, Faculty of Health Sciences, Oslo Metropolitan University, Oslo, Norway; 6 Department of Internal Medicine, Kathmandu University School of Medical Sciences, Dhulikhel, Nepal; 7 Department of Health and Caring Sciences, Faculty of Health and Life Sciences, Linneaus University, Kalmar, Sweden; 8 Department of Obstetrics and Gynecology, St. Olavs University Hospital, Trondheim, Norway; 9 Center for Women’s, Family and Child Health, Faculty of Health and Social Sciences, University of South-Eastern Norway, P.O. Box 235, N-3603 Kongsberg, Norway

**Keywords:** Antenatal care, Domestic violence, Pregnancy, Randomized controlled trial, Nepal

## Abstract

**Background:**

Domestic violence (DV) prior to, and during pregnancy is associated with increased risks for morbidity and mortality. As pregnant women routinely attend antenatal care this environment can be used to offer support to women experiencing DV. We have developed a video intervention that focuses on the use of behavioral coping strategies, particularly regarding disclosure of DV experiences. The effectiveness of this intervention will be evaluated through a randomized controlled trial (RCT) and a concurrent process evaluation.

**Methods:**

All pregnant women between 12–22 weeks of gestation attending routine antenatal care at two tertiary level hospitals in Nepal are invited to participate. DV is measured using the Nepalese version of the Abuse Assessment Screen (N-AAS). Additionally, we measure participants’ mental health, use of coping strategies, physical activity, and food security through a Color-coded Audio Computer Assisted Self Interview (C-ACASI). Irrespective of DV status, women are randomized into the intervention or control arm using a computer-generated randomization program. The intervention arm views a short video providing information on DV, safety improving actions women can take with an emphasis on disclosing the violence to a trusted person along with utilizing helplines available in Nepal. The control group watches a video on maintaining a healthy pregnancy and when to seek healthcare. The primary outcome is the proportion of women disclosing their DV status to someone. Secondary outcomes are symptoms of anxiety and depression, coping strategies, the use of safety measures and attitudes towards acceptance of abuse. Follow-up is conducted after 32 weeks of gestation, where both the intervention and control group participants view the intervention video after completing the follow-up questionnaire. Additionally, a mixed methods process evaluation of the intervention will be carried out to explore factors influencing the acceptability of the intervention and the disclosure of DV, including a review of project documents, individual interviews, and focus group discussions with members of the research team, healthcare providers, and participants.

**Discussion:**

This study will provide evidence on whether pregnant women attending regular antenatal visits can enhance their safety by disclosing their experiences of violence to a trusted person after receiving a video intervention.

**Trial registration:**

The study is registered in ClinicalTrial.gov with identifier NCT05199935.

## Introduction

Domestic violence (DV) has been defined as any form of physical, mental, sexual and/or economic abuse perpetrated by an individual against another person with whom they share a family relationship [1]. DV has a broader specter of possible perpetrators compared to intimate partner violence (IPV) which is limited to the current or former partner [2]. While the intimate partner is the most common perpetrator of DV, others such as in-laws have been identified [3–5].

Women typically report a lower prevalence of violence during pregnancy compared to the violence they report having experienced 12 months prior to pregnancy and/or their lifetime exposure [5–7]. A recent systematic review on intimate partner violence in pregnancy, including 150 studies from over 50 countries reported that the global prevalence of any type of intimate partner violence in pregnancy was 25% [8]. Prevalence figures were higher in Africa (36%) than in Asia (32.1%), South (25.6%) or North America (20.4%). The lowest prevalence was reported for Europe (5.1%). When measured, emotional/psychological violence is usually the most common form of violence, followed by physical violence with the lowest rates found for sexual violence. Two recent studies in Nepal among pregnant women reported lifetime DV from 21 – 27.7% [5, 7].

DV during pregnancy has been associated with miscarriage, late entry into prenatal care, fewer antenatal visits, inadequate weight gain during pregnancy, preterm birth, low birthweight, longer postpartum hospitalization, early cessation of breastfeeding, postpartum depression, and perinatal death [9–13].

Pregnancy presents a significant opportunity to address DV, considering the regular contact in this period between women and healthcare providers [14–16]. Despite limited evidence of its efficacy, many countries have implemented routine inquiry for the experience of violence during pregnancy. However, there have been challenges in implementation of routine inquiry, including reluctance among health professionals [17]. Other barriers to asking about violence in antenatal care include lack of training, time constraints, and insufficient options for assistance and referrals following disclosure [17]. In Nepal, routine inquiry about violence in antenatal care has not been implemented.

However, Nepali health professionals have gained awareness of the problem and possible consequences of DV due to initiatives by national governments, non-governmental organizations and national and international research projects [16, 18–21]. In contrast, pregnant women attending antenatal care may lack knowledge about the scope and risks associated with DV as well as available courses of action they can take. Some women may perceive DV as normal and acceptable under certain circumstances [22].

To cope with violence, women apply both emotion-focused strategies (e.g. use of religion, placating the husband, denial, self-blaming) and problem-focused strategies (e.g. seeking support from formal institutions and social networks) [23]. One such problem-focused coping strategy is seeking social support by disclosing about the difficulties one is experiencing [23]. Studies have investigated the effect of providing pregnant women experiencing DV with problem-focused coping strategies including disclosure of violence to a trusted person [21, 24, 25]. Evidence indicates that women will use more and new coping strategies after being given information about them [21, 25].

In previous research led by our study collaboration, we carried out a pilot study in antenatal care in Nepal which informed women of the different forms that DV can take as well as safety increasing activities, using a flipchart [21]. While the results were promising, using a flipchart is time consuming and difficult to implement in routine antenatal care. Thus, in this phase of the study we have transferred the flipchart information to the format of a short video. This allows women to receive the information in private when using a headset and tablet computer and removes some barriers to health professionals providing this information, such as lack of skills. To gain high quality evidence, we are conducting a randomized controlled trial (RCT) testing the effect of the intervention video in antenatal care, the Addressing Domestic Violence in Antenatal Care Environments RCT (ADVANCE RCT). This protocol paper describes the design of the trial and provides a rationale for the elements included in the study.

## Hypothesis and objectives

The aim of the ADVANCE RCT study is to assess the effect of an intervention video in antenatal care on the use of behavioral coping strategies in relation to the experience of DV, in particular disclosing about the experience.

The null hypothesis is that there is no difference in the proportion of women who disclose someone between the women who view the intervention video compared to the women who view the control video, at baseline.

The primary aim of the process evaluation is to investigate the factors that influence the acceptability of the video intervention and the disclosure of DV. Additionally, the evaluation seeks to examine the various contexts, individual agency, and stages involved in women’s help-seeking behaviors.

## Methods: participants, intervention and outcomes

### Study setting

The study is conducted at two non-governmental hospitals in Nepal, Dhulikhel Hospital (DH) and Kathmandu Medical College (KMC). DH is a community tertiary center situated in Dhulikhel, east of Kathmandu. KMC is located centrally in Kathmandu. At DH, routine antenatal care for low-risk women is provided by nurse-midwives. The rest of the women at DH are cared for by obstetricians. At KMC all antenatal care is provided by obstetricians.

### Eligibility criteria and recruitment

All women attending routine antenatal care at DH or KMC, aged 18 or more, with a pregnancy between 12 – 22 weeks gestational age are invited to participate by study staff. Initially, women are informed that the study seeks to improve reproductive health and involves completing two questionnaires and watching a video twice. Women expressing interest to participate further are then taken to a private room where it can be safely explained that the study includes questions about violence and their formal consent to participate is requested. Consenting women participate in the study by completing a questionnaire and watching the video using an electronic tablet equipped with a headset. Women unable to read can use a data capture method known as a Color-coded Audio Computer Assisted Self Interview (C-ACASI) to give consent and complete the questionnaire, as described in our previous study [21]. All women who complete the questionnaire at the first time point (questionnaire 1) will receive a phone call around 32 weeks gestation to invite them to complete a follow-up questionnaire (questionnaire 2).

**Fig. 1:**
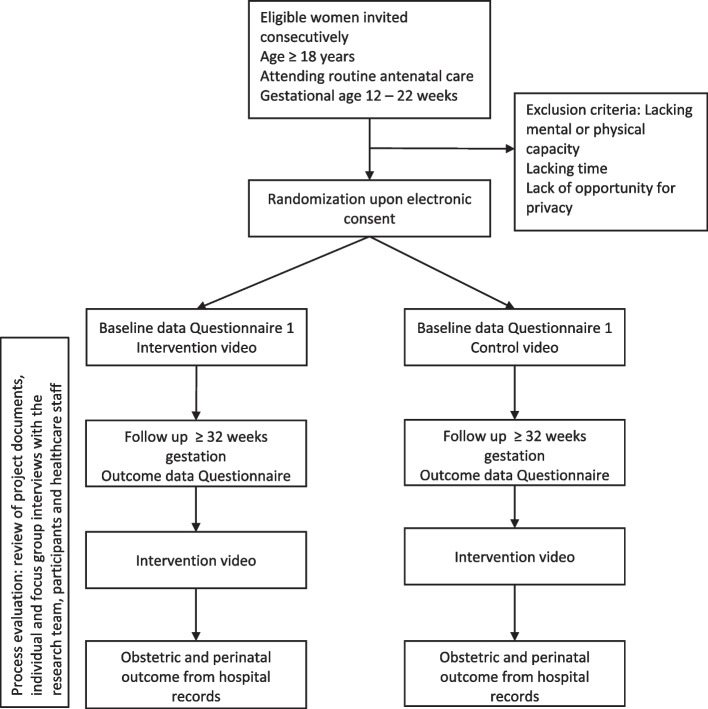
The study flow chart

The schedule of enrollment, intervention and data collection is displayed in Fig. 1.

### The intervention

The intervention video is a further development from the flipchart intervention in our pilot study [21]. The video lasts a total of 7 min and starts with a presentation of what violence is. To tell the story the video uses two fictive persons who tell about their experience of violence. In this way physical, emotional and sexual violence are presented using examples. The video informs women that they are not alone in this experience. The national helpline number and shelters are mentioned. It subsequently presents the same safety promoting activities (i.e. problem-focused coping strategies) as used in the pilot study including disclosing about the experienced violence [21]. Women are told that it is not their fault that they are experiencing the violence. The list with suggested activities and number for the helpline is repeated. The video uses the drawings from the flipchart that have been animated as well as some new ones. The whole video is texted.

The control video also lasts 7 min and presents warning signs and symptoms women can experience during pregnancy that are associated with complications and risks for adverse pregnancy outcomes. This is a repetition of information provided during antenatal care sessions. Permission to use this video has been granted by MedicalAidFilms who produced it. This film was produced to raise community awareness to promote a healthy pregnancy [26]. It too uses drawings and is texted in Nepali.

All women, irrespective of randomization arm receive a leaflet produced by the Nepal Health Authorities that promote a healthy pregnancy and informs women when to contact medical services.

### The exposure

A culturally- and linguistically- relevant version of the Abuse Assessment Screen is used to measure the experience of DV [24, 25, 27]. The original Abuse Assessment Screen for DV was developed in the USA [27], and has been widely used both clinically and in research [24, 28]. The Nepalese-version of the screening tool has been validated by our team (publication forthcoming).

### Outcomes

The primary outcome is the proportion of women who disclose someone outside the research team about their experience of DV. The outcome questions are common across both questionnaires to maximize comparison of data between the two time points.

Secondary outcomes are:

1. *Anxiety and depression*: The ten-item Hopkins Symptom Checklist (HSCL-10) is used to measure symptoms of anxiety and depression. HSCL-10 demonstrates good sensitivity and specificity for detecting mental distress and is widely used, also in the Asian region [29, 30]. Out of 10 items, 6 indicate symptoms of depression and four symptoms of anxiety. For each item the answering options range from not at all (1) to extremely (4). The rationale for measuring distress is twofold. Firstly, we presume that women hearing that violence is not their fault, that they are not alone, that it is not acceptable and that there are possible actions to be taken may become less distressed. Secondly there is the expectation that women who disclose someone about the violence will experience support and thus less distress.
2. *Coping strategies*: The use of coping strategies will be measured using the Ways of Coping Checklist (WCCL) selecting items from the subscales measuring seeking social support (7 items), detachment (6 items) and self-blame (3 items) [31, 32]. Assessing coping strategies using similar questions has been done in Nepal in several studies [33, 34]. The response format is on a 4-point Likert scale, where 0 = does not apply and /or not used, 3 = used a great deal. We include these scales as we hope the information in the video will reduce the use of self-blame and detachment strategies and increase the use of support seeking strategies.
3. *Safety measures*: The use of specific safety measures in relation to living with DV is measured using the list developed by McFarlane et al. (2002) which we modified to the Nepali setting in our pilot study [21, 25].
4. *Attitudes towards abuse*: Attitudes towards acceptance of abuse will be measured using 5 questions used by the WHO [35] and national health survey in Nepal [36]. In addition will we include 6 items from the Social Acceptance of Wife Abuse Scale (SAWAS) which cover other situations and actions known to be found an acceptable reason for violence [37]. The video tells women that they are not to blame for the abuse they receive. Thus, no actions on a woman’s part justify the use of violence.

### Background and associated variables

We collect data on age, education, and income for the woman and her partner, if she has one. We also collect data on ethnicity, if they are living rurally or in a city and if they are living in a joint family or not. We also collect data on food insecurity using Household Food Insecurity Access Scale previously used in Nepal [38, 39]. Food insecure pregnant women are more likely to suffer from violence from her household members [40]. Physical activity (in relation to nutritional status) will be measured by modifying questions from the Global Physical Activity Questionnaire [41]. Suffering from violence may have led to unintended pregnancy [42] so we collect data on pregnancy intendedness using London Measure of Unplanned Pregnancy Scale [43]. These background and associated variables can potentially influence both the exposure and the outcomes.

### Sample-size

In this study we will include 2000 pregnant women. The sample-size calculation is based on the findings from the national Demographic and Health Survey in Nepal 2016 and the validation study of the Nepal Abuse Assessment Screen (N-AAS) done in preparation for this study (publication forthcoming). The most recent Nepal survey at the time of planning the study reported that, among women who had experienced physical and sexual violence, 34% had talked to someone about it [36]. The validation study showed that 12% of the pregnant women reported the experience of DV (publication forthcoming). To detect an increase of 20 percentage points from 35 to 55% in the proportion of women who talk to someone about their experience of DV, the recruitment of 240 women experiencing DV will give at least 80% power with a significance level of 5% and after a presumed 15% attrition rate. With an expected prevalence of 12% this requires the recruitment of 2000 women.

### Randomization

The randomization on a 1:1 basis will be in blocks of 16. The randomization is computer generated, by recruitment site. The randomization program is installed by an external programmer. Upon consenting to participate in the study, women will be allocated into the intervention or control group irrespective of their DV status.

### Blinding

Healthcare staff caring for the women are blinded to the allocation as long as the woman does not discuss the content of the video with them. The statistician who will perform the analyses for the outcome paper is blinded to the allocation. Women are blinded to the allocation as the recruitment room has been organized in such a way that women do not view other screens than their own. Recruitment staff, however, are not blinded to the allocation of the women and participants may consult them about their own situations and require referrals.

### Process evaluation

The process evaluation will involve various participants, including 20–25 women who took part in the RCT, approximately 24 healthcare providers through Focus Group Discussion (FGD) and health managers from the gynecology and obstetrics departments. Additionally, interviews will be conducted with research assistants and doctoral researchers who are involved in the study following the guidelines of the Medical Research Council (MRC) framework [44] which includes considerations such as adaptations, reach, fidelity, and dosage.

The main outcome of the process evaluation is an assessment of the acceptability of the video intervention, specifically investigating the various factors that influence acceptability, such as the time required to explain and watch the video and safety considerations for the women involved. Additionally, the process evaluation will explore the factors that impact women’s disclosure of DV. This includes exploring their agency in seeking help, the relevance of the intervention content in facilitating effective assistance, and the different stages they experience during the process of seeking support for DV.

## Methods: data collection, management, and analysis

### Data collection

Participants who provide consent will complete a questionnaire using an electronic tablet. The questionnaire design has been adapted to accommodate and support women who have reading difficulties. The C-ACASI method enables women to listen to the questions and provide color-coded answers [45, 46]. For women who do not require this assistance, they have the option to read and answer the questionnaire directly without listening to the questions, which can expedite the process for them. Women complete one questionnaire at baseline (Q1) and one approximately 10–26 weeks later (Q2). For completing the follow-up questionnaire, women will receive a phone call from the research assistant to confirm their previous participation in the study and extend an invitation for a second time during their regular antenatal visit. Additionally, research assistants will gather data from the patients’ obstetric notes, including both the antenatal card and hospital register.

### Analysis

A detailed statistical analysis plan (SAP) will be developed.

The main outcome is measured by comparing the proportion of women in the intervention and control groups who have told someone about the violence they have experienced. The comparison will be presented as risk ratios with 95% confidence intervals. In addition, we will collect data on whom the woman has told about the violence and will present this as descriptive findings. A detailed description of the analysis strategy for the secondary variables will be provided in the SAP. Briefly, binary outcomes will be compared between intervention arms using risk ratios, and continuous secondary outcomes will be compared using linear mixed models to take into account the baseline values of each outcome variable. Non-parametric analysis or alternative regression models will be considered where the assumptions for the linear regression models are not met.

### Process evaluation

The observations of the RCT and individual in-depth interviews with participating pregnant women will be conducted at different stages during the implementation of the RCT and upon its conclusion. The FGDs with healthcare providers and health managers will take place at the end of the RCT, while interviews with research assistants and doctoral students will occur after the analysis of RCT outcomes. The interviews will be recorded, transcribed, and analyzed using inductive coding to derive themes, theories, and concepts from the data, thereby revealing underlying structures of experiences or processes [47].

Data management and analysis will follow a convergent parallel mixed method design. Qualitative and quantitative data will be analyzed separately and then merged and integrated at the interpretation level. Data triangulation, including the use of a triangulation matrix and discussions with co-researchers, will be employed to ensure a comprehensive analysis.

### Adverse events and data monitoring

The study will be supervised by an internal Data Safety and Monitoring Committee (DSMC). A safety protocol has been set up and adverse events will be reported to the committee as they occur. The safety protocol includes immediate referral opportunities for women depending on their individual needs. The characteristics of the trial are such that an external data monitoring and safety committee was considered unnecessary. These characteristics include a low risk of harm to the participants due to the trial and negligible credibility/integrity concerns about the potential impact [48]. No interim data analyses are planned. The DMSC will monitor the quality of the data.

### Study management

The study is managed by the Principal Investigator (PI) and co-PI in Norway and a local PI at each of the participating hospitals. This team is responsible for ensuring the study is conducted according to the protocol and ethical approval, that women’s privacy is protected, and that women in need of referral or other support receive this. Data-management is regulated by collaborative standard contracts.

### Dissemination

A dissemination event will be organized in Nepal where findings of study will be presented. This event will be open to all stakeholders. The video will be handed over to the National Health Education, Information and Communication Centre at the Department of Health Service, Nepal. We will explore rural uptake of this intervention during the dissemination event. All papers will be submitted for publication in international open access scientific journals.

## Discussion

This pragmatic RCT aims to determine whether pregnant Nepali women, attending routine antenatal care and experiencing DV, are more likely to disclose their experiences after watching an intervention video that encourages disclosure, compared to women who view a control video. Disclosing DV is just one of several safety-promoting behaviors identified for women living with DV [21, 25].

Disclosing the experience does not necessarily mean preparing to leave the situation, such as packing a bag, hiding money, or making copies of important documents. Instead, sharing the experience of DV with someone else allows the person receiving the information to offer support through active listening and acknowledging the challenging situation the woman is facing. Sharing the experience of DV also contributes to breaking the taboos surrounding violence that occurs within the confines of a home.

Furthermore, sharing the experience helps individuals understand the severity of the situation and may prompt the woman to consider her options for action [21, 49, 50]. The nature of support received depends on whom the woman chooses to disclose to. We assume that women carefully assess whom they confide in, aiming to minimize the risk of increased violence. A woman might confide in a female friend who can provide little more than a listening ear and empathy, or possibly share her own experience to let both women know they are not alone. Alternatively, a woman may choose to disclose to a healthcare professional, which could result in a referral for counseling or assistance from an appropriate person or institution.

While our study focuses on the disclosure outcome based on previous research, the process evaluation serves to investigate the indirect effects that may not be directly captured by our quantitative measures. By incorporating a process evaluation, we can gain a more comprehensive understanding of the intervention’s overall impact and its potential to facilitate disclosure and other meaningful help-seeking and support for women experiencing DV. The intervention tested in our study is straightforward and can be easily integrated into routine antenatal care without the need for additional highly qualified staff. The intervention is relevant for all women, including those who have not disclosed experiences of violence. Non-disclosure may stem from women not yet being prepared to acknowledge their situation to themselves or others. Women who are not experiencing violence can gain valuable insights into the experiences of others and learn about helpful actions they can take, potentially using this knowledge to support others.

### Strengths and limitations

This study has several strengths. First, we have conducted prior investigations on the experiences, needs, and expectations of women living with DV from health services during pregnancy [51]. Secondly, we have explored women's perceptions of DV [22]. Thirdly, we conducted a pilot study to test the presentation of safety behaviors related to DV experiences among women receiving routine antenatal care, regardless of their disclosure of such experiences [21]. Fourthly, we have tested the use of the C-ACASI method for data collection at the same recruitment sites as in the current study [21].

These previous studies have allowed us to identify areas for improvement in the current intervention. Moreover, these studies led us to validate the Abuse Assessment Screen for the Nepali setting before commencing the proposed RCT.

One limitation of our study is that we have not found a practical solution for repeating the intervention multiple times before assessing the outcome. Ideally, we would like women to view the video several times or at least have the option to view it again upon request. Due to study setting and staffing constraints, this has not yet been feasible. Nevertheless, we believe that women experiencing DV may be more receptive to the video's content compared to women without such experiences. Therefore, even a single viewing is expected to have an impact on women's lives. In addition, it is possible that women may discuss and share the content of their video with other women participated in the study leading to contamination.

### Status and timeline of the study

The study began enrolling participants on the 29^th^ January 2023, with a planned inclusion period spanning 16 to 20 months. The end of the trial will be determined once the required total number of women who have experienced violence and have responded to the second questionnaire has been reached.

## Data Availability

Not relevant at this stage.
